# Ultrastructural Exploration on the Histopathological Change in *Phenacoccus fraxinus* Infected with *Lecanicillium lecanii*

**DOI:** 10.1371/journal.pone.0117428

**Published:** 2015-01-28

**Authors:** Ying Gao, Ying Ping Xie, Qi Xiong, Wei Min Liu, Jiao Liang Xue

**Affiliations:** 1 School of Life Science, Shanxi University, Taiyuan, China; 2 Institute of Applied Biology, Shanxi University, Taiyuan, China; UMR INRA/INSA, BF2I, FRANCE

## Abstract

The histopathological changes of the second instar nymph of the mealybug *Phenacoccus fraxinus* infected with *Lecanicillium lecanii* strain 3.4505 were investigated using light, scanning and transmission electron microscopy. The results demonstrated that *L. lecanii* 3.4505 could infect *P. fraxinus* in a short period. At 24 h post-inoculation, the conidia of *L. lecanii* 3.4505 adhered to the indented gloves or intersegmental folds of the insect body surface. Subsequently, the germinated conidia produced germ-tubes, appressoria and extended hyphae, which tightly adhered to the cuticle. Penetration of cuticle could be achieved either by peg form appressoria or directly by hyphae. Also, the conidia and hyphae could secrete massive mucilages causing visible damage to the host cuticle. After 48 h, the body wall, tissues and organs, including cuticle, trachea, fat body, muscle, Malpighian tubules and nerve ganglion, were destroyed by ramification of hyphae as a result of infection. The endoplasmic reticulum hypertrophied and formed obvious fingerprint agglomerates, and the mitochondria swelled and deformed in the haemocytes. Finally, the mycelium fully occupied the entire haemocoel. The entire bodies were wrapped in a white mycelium, with the mycelium extending radically outward.

## Introduction

The mealybug *Phenacoccus fraxinus* Tang (Hemiptera: Pseudococcidae) is an important pest on the ash tree *Fraxinus* spp., causing rampant harm in several provinces and cities in China and seriously affecting the growth of trees and city landscapes [1]. The control effect of chemical pesticides is restricted because this insect secretes abundant protective wax. Furthermore, pesticides cause environmental pollution and injure the natural enemies of pests. Biological control is a potentially important means, and the application of entomopathogenic fungi would be highly desirable in the wider natural ecosystems.

*Lecanicillium lecanii* is a well-know entomopathogenic fungus [2]. It was originally isolated from *Lecanii coffeae* Walker, in Ceylon by Nivter in 1861. In recent years, most studies concerning this pathogen have focused on its use for the control of aphids, whiteflies, and mites. However, *L*. *lecanii* has not been reported in the biological control of *P*. *fraxinus*. Moreover, the infection mechanism of *L*. *lecanii* on *P*. *fraxinus* is not known. In the present study, we investigated the infection process and histopathological changes of *P*. *fraxinus* infected by *L*. *lecanii*. The results of this research will provide support for this fungal application as a potential biological control agent.

## Materials and Methods

### Ethics Statement

The collection of the second instar nymphs of *P*. *fraxinus* was permitted by the Bureau of Parks and Woods of Taiyuan County, Shanxi Province, China.

### Entomopathogenic fungus and test insects

The second instar nymphs of *P*. *fraxinus* were collected from the ash trees *Fraxinus chinensis* Roxburgh (Oleaceae) at Taiyuan city (E112˚53ˊ, N37˚87ˊ) in Shanxi Province in China. One hundred individuals of the living nymphs were used for the experiment. The entomopathogenic fungus *L*. *lecanii* strain 3.4505 originally isolated from a species of scale insect was purchased from China General Microbiological Culture Collection Center, and was selected for the trial. The suspension concentration was 5 × 10^7^ spores/mL. The methods of fungal cultivation, conidial harvest and of insect inoculation were previously described by Liu [3].

### Observation of external symptoms

The external symptoms of the scale insects infected with the fungus were directly observed at 24, 48, 72, and 96 h post inoculation using a stereomicroscope (OLYMPUS SZ-ST). The infection characteristics and the number of infected insects were recorded, and the photographs were taken using an Olympus C5050Z digital camera (OLYMPUS OPTICAL Co., Ltd).

### Histopathological observations

**Sample fixation**. At 24, 48, 72, and 96 h post inoculation, approximately 20 scale insects for each observation period were collected and immersed in 4% (v/v) glutaraldehyde (pH 7.2, 0.2 M phosphate buffer) for 48 h at 4°C, respectively. After rinsing thrice with 0.2 M phosphate buffer, the samples were ready for further processing for light microscopy (LM), scanning electron microscopy (SEM) and transmission electron microscopy (TEM).

**LM observation**. The rinsed samples were gradually dehydrated in a series of ethanol solutions [35%, 55%, 75%, 85%, 95%, and 100% (v/v)] and xylene infiltrated (35%, 55%, 75%, 85%, 95%, and 100%), for 10 min at each level. The samples were embedded in xylene: paraffin 1:1(v/v) for 48 h at 56°C, transferred in paraffin for 48 h at 56°C. The embedded specimens were serially sectioned into 6 μm slices using Reichert HistoSTAT820. The sections mounted on glass slides were mordanted with 2% ferrovanadium (30 min), stained with 0.5% hematoxylin (1.5 h), and then destained with saturated picric acid (1.5 h). Finally, the slides were enclosed in neutral balsam and observed under an OLYMPUS BX-51 LM (OLYMPUS Co., Ltd., Japan). Photographs were taken with an Olympus-C5050Z digital camera.

**SEM observation**. The rinsed samples were dehydrated in a gradient of acetone solutions (10% to 100%) for 10 min at each level. The samples dried using supercritical drying apparatus EMS 850 were fixed on microscope slides and sputter-coated with gold about 20 μm and observed using a SEM (JSM-840 model JEOL Ltd., Japan) operated at 15 kV. Micrographs were taken using a Canon EOS 350D digital camera.

**TEM observation**. The samples for TEM were post-fixed in 1% (v/v) osmium tetroxide (in phosphate buffer) for 3 h at 4°C, dehydrated in an ethanol series (10% to 100%), and embedded in Epon 812. Semithin sections (1 μm) were mounted on glass slides and stained with 1% (v/v) toluidine blue and observed using a LM (Olympus BX-51). Ultrathin sections (0.08 μm) were cut using a Reichert Jung ultramicrotome, collected on copper grids, and counterstained with uranyl acetate and lead citrate. The ultrathin sections were observed using a TEM (JEM-1200EX, accelerating voltage 80 kV). Micrographs were taken on Lucky film and they were scanned using a N-TEK Nuscan 700 scanner.

## Results and Discussion

### External characteristics of hyphae on the surface of the host

**Fig 1:**
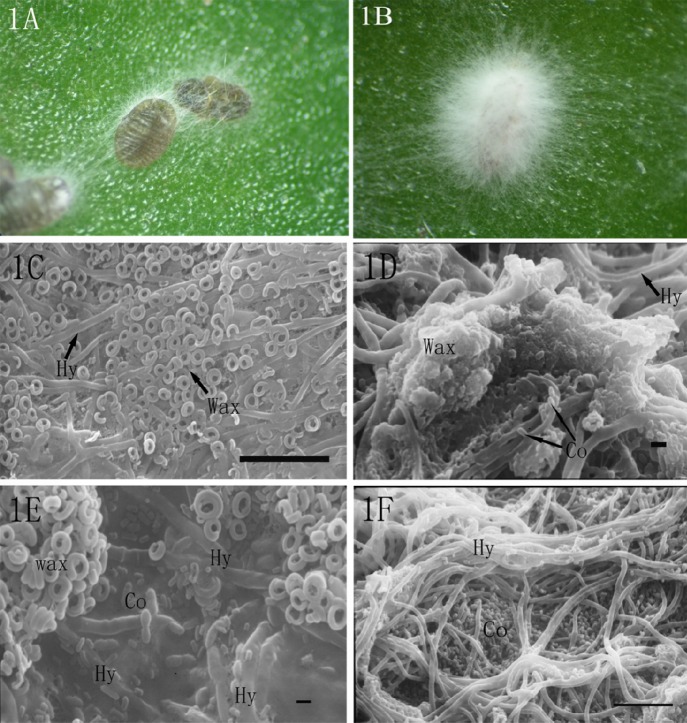
Hyphae on the nymph body surface. (1A) The hyphae grew around the body marginal regions. (1B) The body of nymph was completely covered by white mycelium. (1C) The hyphae (Hy) passed through the waxy filament (3000×, bar = 10 μm). (1D) The hyphae (Hy) passed through the wet waxy agglomeration (4000×, bar = 1 μm). (1E) The intersegmental folds were invaded by the conidia (Co) and by the hyphae (Hy) (5000×, bar = 1 μm). (1F) Many spores scattered among the hyphae (Hy) (2000×, bar = 10 μm).

*P*. *fraxinus* can generate and excrete various wax substances on its body surface [4]. The observation showed that, despite the wax protection, *L*. *lecanii* strain 3.4505 could successfully infect the insects. There were some hyphae on the body surface, particularly around the body marginal regions at 24 h post-inoculation (Fig. 1A). The hyphae grew so rapidly that the white mycelium completely covered the insect body at 96 h post-inoculation, and the insect bodies were shriveled (Fig. 1B). Observation under scanning electron microscope revealed that the hyphae could easily pass through the waxy filaments (Fig. 1C) and even through the wet waxy agglomeration (Fig. 1D). The intersegmental folds were also easily invaded, with many conidia adhering and producing hyphae (Fig. 1E). In a magnified view, many spores scattered among the mycelia (Fig. 1F). Compared to *Ceroplastes* *japonicus*, the hyphae appeared earlier and grew more rapidly on the nymphs of *P*. *fraxinus*[3]. For example, the nymphs of *P*. *fraxinus* were completely covered with thick hyphae at 96 h post-inoculation while 144 h post-inoculation were necessary in *C*. *japonicus*. This difference could be due to the marked characteristics and ultrastructural difference of the wax filaments of *P*. *fraxinus* compared with those thick wax textures of *C*. *japonicus* [4,5]. Additionally, the inhibition of the wax filaments to hyphae invasion in the former was less than that of the thick wax texture of the latter.

### Fungal adhesion and infection on the host surface

**Fig 2:**
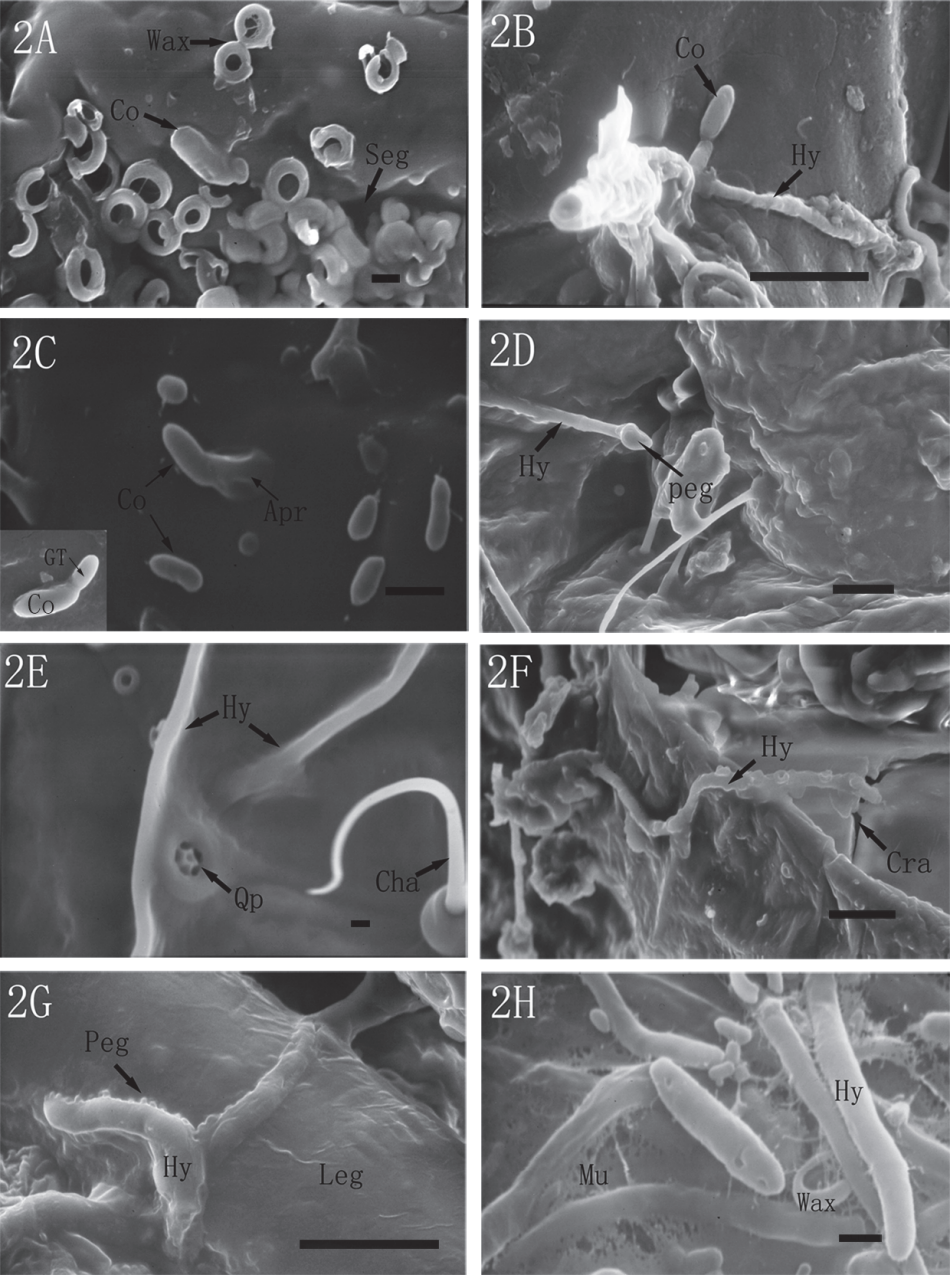
SEM micrographs of the attachment and invasion of *L. lecanii* on the surface of *P. fraxinus*. (2A) Few conidia (Co) attached onto the intersegmental fold (If) (6000×, bar = 1 μm). (2B) Conidia (Co) adhered onto the body grooves (3000×, bar = 10 μm). (2C) Conidia (Co) produced germ-tube (GT) and appressorium (Ap) (15000×, bar = 1 μm). (2D) A specialized cone penetration peg (1500×, bar = 10 μm). (2E) Hypha (Hy) vertically penetrated the cuticle of the scale insect. Qp: quinquelocular pores, Cha: cheta (4000×, bar = 1 μm). (2F) Hypha (Hy) directly penetrated into the haemocoel through cracks (Cra) (1600×, bar = 10 μm). (2G) Hyphae (Hy) produced some small and dense penetration pegs on the leg of the scale insect (3500×, bar = 10 μm). (2H) Hyphae (Hy) produced copious mucilage (Mu) (10000×, bar = 1 μm).

Under scanning electron microscopy, at 24 h post-inoculation, few conidia were observed on the intersegmental folds (Fig. 2A) and in grooves of the body surface (Fig. 2B). The conidia germinated within 24 h after inoculation and subsequently produced germ-tubes and appressoria. In Fig. 2C, one germinated conidium showed a comparatively short germ-tube, and another conidia directly produced an appressorium-like structure, which tightly adhere onto the cuticle. Some of the germ-tube continued growing and developed into hyphae. During the growth period, a specialized cone-shaped appressorium was produced at the end of the hyphae (Fig. 2D). The elongated hypha directly penetrated the cuticle of the scale insect (Fig. 2E). With the action of hyphae during the infection, some cracks were produced on the cuticle. As shown in Fig. 2F, a hypha directly penetrated into the cuticle via a crack. On the leg of the scale insect, massive mucilage was secreted by the hyphae, which in turn might strengthen the adhesion and penetration due to the cuticle degrading enzymes they contain (Fig. 2G-H).

The mode of fungal infection of a host is primarily via cuticle invasion [6], which is in agreement with the present study. Similar observations were also made by [3] on *L*. *lecanii* against *C*. *japonicus*. Additionally, the characteristics of the conidia adhesion, germination, hyphae growth and penetration are similar to the ones observed on *C*. *japonicus* infected by *L*. *lecanii* [3]. However, in the present study, our investigation clearly showed that the hyphae spread on the insect host cuticle often coated with massive dense mucilage, which might contain extracellular cuticle degrading enzymes, e.g., proteinase, chitinase and lipase that may help the entomopathogen to dissolve and penetrate the cuticle. Some other entomopathogens, i.e., *Metarhizium anisopliae*, *M*. *flavoviridae* and *Beauveria bassiana*, have been shown to secret an array of similar lytic enzymes [7, 8]. Our previous study showed that the activities of extracellular protease and chitinase from *L*. *lecanii* significantly increased when the body materials of scale insects were added into PDA medium, and their activities had a linear correlation with the mortalities of the scale insects [9]. Another study indicated that the protease secreted by *L*. *lecanii* played important roles in decomposing the cuticle protein and in exposing chitin during the infection against the scale insect [10].

### Integument penetration

**Fig 3:**
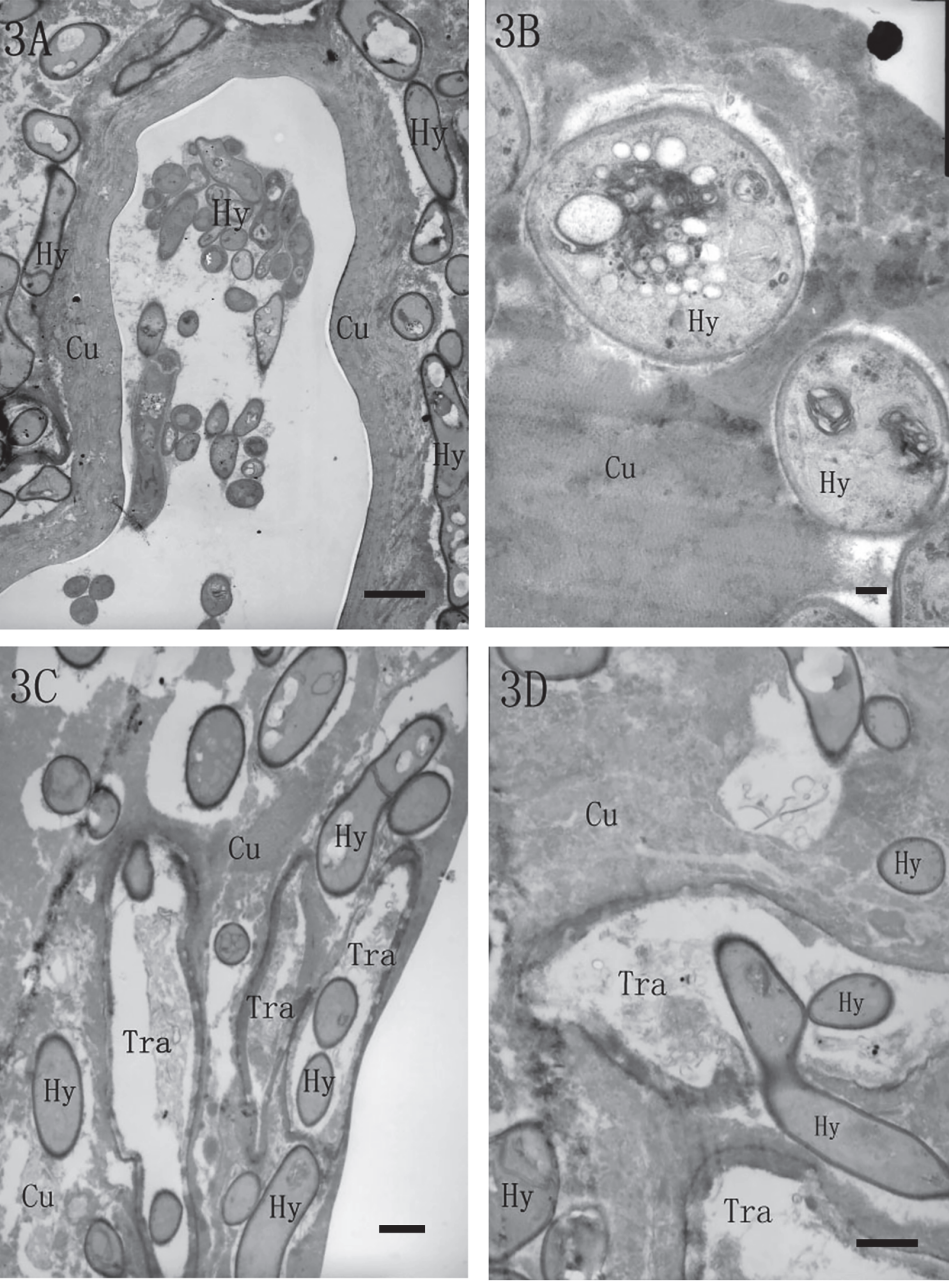
TEM micrographs of hyphae that penetrated the integument. (3A) Many hyphae (Hy) aggregated in the intersegmental folds, and many hyphae colonized inside the cuticle (Cu) (4000×, bar = 2 μm). (3B) The procuticle structure was destroyed and disappeared near the hyphae (Hy) (20000×, bar = 200 nm). (3C) Many hyphae (Hy) entered the insect trachea (Tra) in the cuticle (6000×, bar = 1 μm). (3D) Hypha (Hy) penetrating through the tracheal wall in the cuticle (Cu) (8000×, bar = 1 μm).

Under transmission electron microscopy, hyphae aggregated in the intersegmental folds and colonized inside the integument (Fig. 3A). In a magnified view, the procuticle structure was destroyed and disappeared near the hyphae (Fig. 3B). In addition, we first observed many hyphae entering the insect trachea in the cuticle, which resulted in tracheal wall damage (Fig. 3C). In Fig. 3D, one hypha is penetrating through the tracheal wall. The foreside of this hypha entered trachea, whereas the middle part was constricted to make it sharper and easier to penetrate the tracheal wall. Therefore, the pathogenic fungus *L*. *lecanii* could attack the tracheal system of the scale insect by hyphae penetrating the tracheal wall.

### Infection of hemocoel and internal tissues

**Fig 4:**
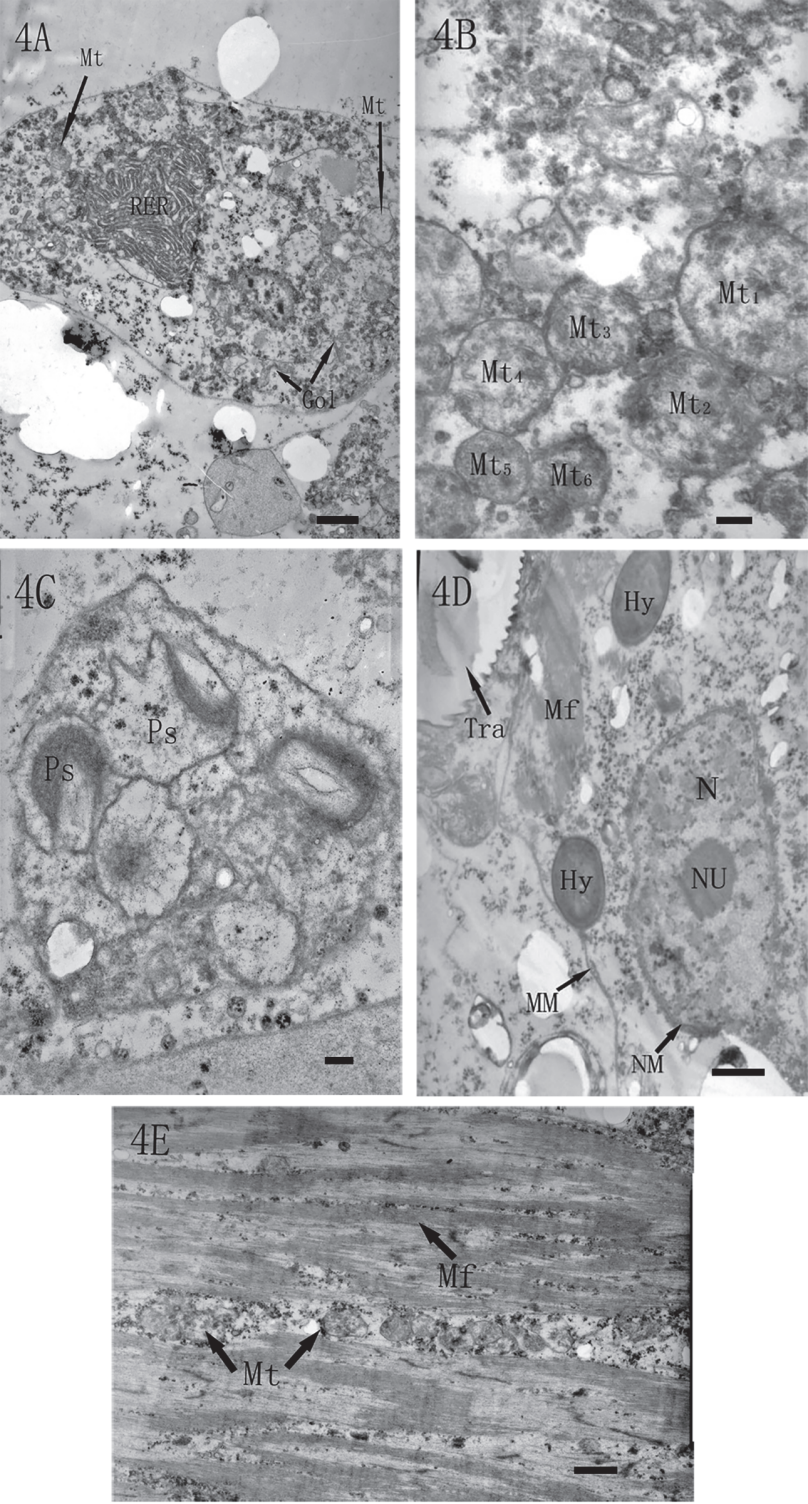
TEM micrographs of the pathologic changes in the hemocoel. (4A) The endoplasmic reticulum (ER) in an infected hemocyte became hypertrophied and agglomerated similar to a fingerprint (4000×, bar = 1 μm). (4B) Infected mitochondria (Mt) appeared swollen, with ruptured membranes and with dissolved cristae (25000×, bar = 200 nm). (4C) Morphologic abnormalities of peroxisomes (Ps) (20000×, bar = 200 nm). (4D) The hyphae penetrated into the muscle and caused its cell membrane (MM) to break into pieces. The nuclei (N) of muscle cells were damaged, with part of the nuclear membrane (NM) disappearing and with the nuclear chromatin slightly condensing and escaping. Hy: hyphae, Tra: trachea (8000×, bar = 1 μm). (4E) The loose and irregular myofibrils, the broken myofilaments (My), and the seriously damaged mitochondria (Mt) (6000×, bar = 1 μm).

After *L*. *lecanii* reached the hemocoel, the hemocytes were seriously destroyed. The endoplasmic reticulum in the haemocytes became hypertrophied and formed obvious fingerprint-like agglomerates (Fig. 4A). The mitochondria swelled (Mt_1_) and deformed (Mt_2_, Mt_6_). Their membranes were ruptured (Mt_2_, Mt_3_, and Mt_4_), and their cristae were destroyed (Mt_1–6_) (Fig. 4B). The peroxisome was deformed and damaged (Fig. 4C).

The muscle tissue was also heavily destroyed. The hyphae penetrated into the muscle and made its cell membranes break into pieces. The cell nuclei were damaged, with part of the nuclear membrane disappearing and with the nuclear chromatin slightly condensing and escaping (Fig. 4D). In a magnified view, the myofibrils were loose and irregular; parts of myofilaments were broken and mitochondria in myocytes seriously damaged (Fig. 4E).

**Fig 5:**
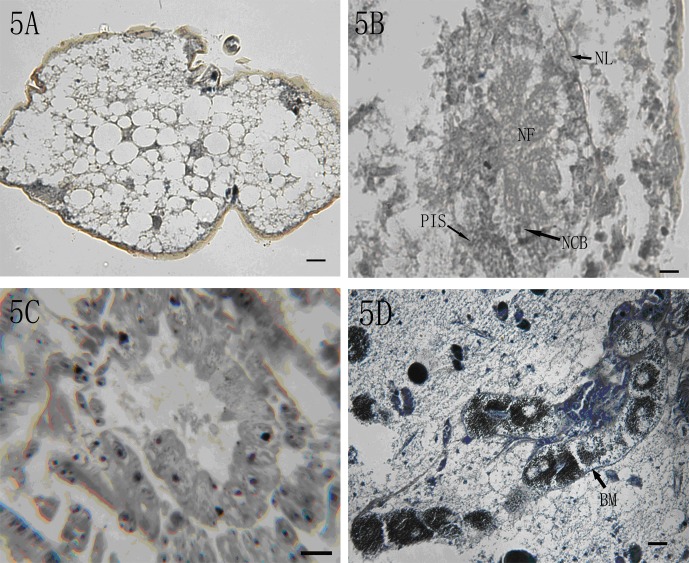
Micrographs of the histopathology of the mealybug. (5A) The normal and damaged fat bodies (200×, bar = 20 μm). (5B) The damaged nerve ganglion, Nl: neural lamella, Pn: perineurium inner sheath, Ncb: nerve cell bodies, Nf: nerve fibers (200×, bar = 20 μm). (5C) The damaged midgut (Mg) (400×, bar = 40 μm). (5D) The damaged Malpighian tubules, BM: basal membrane (200×, bar = 20 μm).

Under light microscopy, fat body cells were abundant in the haemocoel of 2nd-instar *P*. *fraxinus*. The normal fat bodies had clear outlines, whereas the infected fat bodies near the cuticle had dim margins or blurred outlines (Fig. 5A). The nerve ganglion was severely damaged in the neural lamella endo-perineurium sheath, in the nerve cell bodies around the inner edge, and in the nerve fibers in the center (Fig. 5B). Fungal infection in fat bodies and nerve ganglion was observed in *P*. *fraxinus*.

In our study, the normal intestinal cells arranged in order, whereas the damaged intestinal cells were deformed, broken, and even separated from the intestinal muscle layers (Fig. 5C). The basal membrane of the Malpighian tubules separated from the cells, and the compact cell layer was loose and irregular (Fig. 5D). The digestive and excretory systems were destroyed whereas no hyphae were observed inside the intestines. Nevertheless, eventually the pathogen completely colonized all the organs and tissues in the infested host.

## Conclusions

Through the above observations and analysis, the *L*. *lecanii* strain 3.4505 could successfully infect and cause death to the second-instar nymph of *P*. *fraxinus*. The infection process was similar to that observed on *C*. *japonicus*; however, the results indicated that *L*. *lecanii* 3.4505 could easily infect *P*. *fraxinus* in a short time. Tests on the pathogenicity of *L*. *lecanii* against *P*. *fraxinus* in the field need to be carried in the future to support data obtained by ultrastructural observations. This new evidence is meaningful for better understanding the fungal infection of scale insects and for using this fungus as a potential biological control agent.
